# Reviewers in 2023

**DOI:** 10.1098/rsos.240250

**Published:** 2024-03-27

**Authors:** Wendy Hall

**Affiliations:** ^1^

The tradition established by the Royal Society Publishing of publishing an annual list of thanks to peer reviewers who served in the previous calendar year continues in the below article.

The Editors and journal administrative team of *Royal Society Open Science* would like to thank all reviewers who contributed their time by refereeing manuscripts submitted last year. We remain extremely grateful for your support and expertise, and hope you will serve in 2024 and beyond.

Reviewers value recognition of their efforts by the journal and through services such as the Web of Science Reviewer Recognition Service, which is integrated with *Royal Society Open Science*.

To recognise and support our peer reviewers, we list the names of all reviewers who served in 2023 and have opted to be included.

This article is made permanent and citable by its digital object identifier (DOI). We encourage you to quote your contribution in your applications for tenure and grants or other forms of research assessment. Where you have provided it, we have included your ORCID so your contribution can be unambiguously assigned to you.

On behalf of all the Editors and editorial office, thanks once again to all of our reviewers past, present and future, and we look forward to your continued support of our journal.

Ansari MZ

Ayana G

Aalto SL

Aanen D https://orcid.org/0000-0002-5702-1617

Abbasabadi MK

Abboud J

Abd-AlGhafar w https://orcid.org/0000-0003-1489-4239

Aboelalla M https://orcid.org/0000-0002-8579-0093

Abou Chakra M

Abramson J https://orcid.org/0000-0001-7106-6419

Aceto M

Ackerman J

Ackley S https://orcid.org/0000-0003-0181-6063

Aczel B https://orcid.org/0000-0001-9364-4988

Adamczyk PG https://orcid.org/0000-0001-5374-7691

Adamik P

Adegoke A https://orcid.org/0000-0002-2970-2140

Agarwal K https://orcid.org/0000-0002-9762-3014

Ahmad M

Ahn YY

Ajayi OM

Akanyeti O

Akbar Sial Q

Akhtar MN https://orcid.org/0000-0002-0645-0796

Alam K https://orcid.org/0000-0002-3528-0044

Alawsi T https://orcid.org/0000-0002-8221-5405

Albani V

Al-Bazi A

Albert R

Alberts B

Albery G https://orcid.org/0000-0001-6260-2662

Alborn H https://orcid.org/0000-0003-3918-5041

Alejandro FA

Aletta F https://orcid.org/0000-0003-0351-3189

Alexander A

Alexandrovskaya Y

Algermissen J

Allegrucci G

Allen C

Alonso S

Alvarez-Idaboy JR

Amabilino D

Amao A

Andersen H

Andrada El https://orcid.org/0000-0002-9300-4056

Andrews J https://orcid.org/0000-0002-0180-185X

Andrianov G

Angielczyk K

Anglada-Tort M https://orcid.org/0000-0003-3421-9361

Ansaldi M https://orcid.org/0000-0002-2878-0702

Anton S

Aplin L https://orcid.org/0000-0001-5367-826X

Araiza A

Archie E https://orcid.org/0000-0002-1187-0998

Argüello J

Aria AI

Aria C https://orcid.org/0000-0002-0567-1730

Arias MJL

Ariel G

Arnold, P https://orcid.org/0000-0002-6158-7752

Arvanitakis A

Asamoah JKK https://orcid.org/0000-0002-7066-246X

Asgari S https://orcid.org/0000-0001-7885-6544

Ashemary AZ

Ashkenazi S https://orcid.org/0000-0002-4343-4824

Ashwin C

Assaf E

Astegiano J https://orcid.org/0000-0003-0583-7291

Astley H

Asubiaro TV

Athanasiou S

Aubert Bonn N https://orcid.org/0000-0003-0252-2331

Austad S

Averianov A

Ayyappan A

Azarian T https://orcid.org/0000-0002-8611-5241

Azer SA

de Baaij JHF

Baggio J

Bai J

Bai S

Baker-Kukona A

Bakker M

Balshine S

Balthazart J https://orcid.org/0000-0001-9492-2126

Baltzopoulos B

Bandyopadhyay D

Bankova V

Banks C https://orcid.org/0000-0002-0756-9764

Bao Z https://orcid.org/0000-0003-3601-460X

Baquero F

Bara J

Barabadi H

Baragli P

Barak M http://orcid.org/0000-0003-4953-4380

Barban N

Barbeito C

Barker AJ

Barman A

Barnett A

Baron A

Bartlett M https://orcid.org/0000-0002-7391-5135

Bartos L https://orcid.org/0000-0003-2667-5930

Bartoszewski R https://orcid.org/0000-0002-2864-6757

Barve S https://orcid.org/0000-0001-5840-8023

Bassi E

Bauch C https://orcid.org/0000-0001-6214-6601

Bayford R

Beardsworth C https://orcid.org/0000-0003-1308-1455

Beaty R

Bechly G

Becker D https://orcid.org/0000-0003-4315-8628

Beehner J

Beja-Pereira A https://orcid.org/0000-0002-1607-7382

Belyk M https://orcid.org/0000-0002-3270-8666

Benjamin Daniel

Bennett J

Beran M https://orcid.org/0000-0002-2504-0338

Bergman T https://orcid.org/0000-0002-9615-5001

Bertemes-Filho P

Bertolucci C

Bertram J

Besancon L https://orcid.org/0000-0002-7207-1276

Besley J

Bessesen B

Bester-Rogac M

Betti M https://orcid.org/0000-0002-1458-5359

Beukeboom L

Bhattacharjee D

Bhattacharjee S https://orcid.org/0000-0002-6528-6877

Bhattarai A https://orcid.org/0000-0002-2648-4686

Bicca M

Bidula S

Biggs T

Bijleveld E https://orcid.org/0000-0001-5892-3236

Bilde T

Binda P

Bird M

Bitton P-P

Blake K https://orcid.org/0000-0003-4834-4120

Blasco R https://orcid.org/0000-0001-9804-739X

Blázquez J

Blinch J

Blomberg E

Bodala I

Bode N https://orcid.org/0000-0003-0958-5191

Bogaerts L

Bolhassan I

Bolland M

Bonaccorsi G https://orcid.org/0000-0002-5600-6271

Bonani W

Bondarchuk S

Booth W https://orcid.org/0000-0003-2355-0702

Borazjani I https://orcid.org/0000-0001-7940-3168

Bormashenko E https://orcid.org/0000-0003-1356-2486

Bornbusch S https://orcid.org/0000-0001-9996-8369

Bortz D https://orcid.org/0000-0003-1163-7317

Bose A https://orcid.org/0000-0001-5716-0097

Botta F https://orcid.org/0000-0002-5681-4535

Bottrell S

Boudreau B

Bouquet C https://orcid.org/0000-0003-4228-3291

Bourgoin G https://orcid.org/0000-0003-0993-0741

Bouter L

Bower I

Bowes L

Bowles A

Bowman R https://orcid.org/0000-0002-1531-8199

Boyd Z

Branch C https://orcid.org/0000-0002-4671-2420

Brannelly L https://orcid.org/0000-0003-2975-9494

Brascamp EW

Brauner P

Brauwer M

Brazil A

Brennan P

Breuer K

Briefer E https://orcid.org/0000-0003-4147-0319

Briggs C

Brink K https://orcid.org/0000-0001-6630-1525

Brinkman D

Brito P https://orcid.org/0000-0002-4853-8630

Brizi D

Broderick N https://orcid.org/0000-0002-6830-9456

Browne L

Buchan D

Buchanan E https://orcid.org/0000-0002-9689-4189

Bull J https://orcid.org/0000-0002-4373-6830

Burchardt LS https://orcid.org/0000-0002-9210-7934

Burke N https://orcid.org/0000-0002-9843-926X

Burkett J https://orcid.org/0000-0002-6357-5499

Burkhardt E https://orcid.org/0000-0002-5128-4176

Burr D https://orcid.org/0000-0003-1541-8832

Burton AS

Büscher T https://orcid.org/0000-0003-0639-4699

Butler T

Cabanès J https://orcid.org/0000-0001-5900-4135

Cabezas-Cruz A https://orcid.org/0000-0002-8660-730X

Caglayan H https://orcid.org/0000-0002-0656-614X

Calderan S

Camargo C

Cambier N

Campbell D https://orcid.org/0000-0003-4028-8347

Campbell D https://orcid.org/0000-0001-8996-5463

Campbell H https://orcid.org/0000-0002-0959-1594

Campbell M

Campillo-Funollet E

Campos L https://orcid.org/0000-0001-5414-8746

Canini L

Cao W

Cappa F https://orcid.org/0000-0002-3510-8885

Capraro V https://orcid.org/0000-0002-0579-0166

Caputo M

Cardini A https://orcid.org/0000-0003-2910-632X

Careau V https://orcid.org/0000-0002-2826-7837

Carlson K

Carragher D

Carrus E https://orcid.org/0000-0001-9197-8757

Carter E

Caruana M https://orcid.org/0000-0002-2095-5360

Cass J

Castioni P https://orcid.org/0000-0001-5560-7094

Catterson E

Cecilia H

Ceia F

Çelik S

Cenci S https://orcid.org/0000-0001-6843-468X

Cenzer M https://orcid.org/0000-0003-4665-1207

Čerňanský A

Cerrada M https://orcid.org/0000-0003-4379-8836

Chakraborty S

Chakravarty P https://orcid.org/0000-0002-2975-6253

Challis J

Chamberlain R

Chamberlin H https://orcid.org/0000-0001-7203-2691

Chamorro L

Chan HF https://orcid.org/0000-0002-7281-5212

Chan KO https://orcid.org/0000-0001-6270-0983

Chandra V

Charafeddine R

Charrier G

Chaves Dias F

Cheke L

Chen Junning

Chen L https://orcid.org/0000-0002-0394-4387

Chen M https://orcid.org/0000-0001-9663-7441

Chen Z

Chen Z-Q https://orcid.org/0000-0001-5341-6913

Cheng B

Cheng Y

Cheung C-N https://orcid.org/0000-0003-2020-4679

Chiappini N

Chirichella R

Chivite M https://orcid.org/0000-0003-1917-1523

Chodrow P https://orcid.org/0000-0001-5667-490X

Choiniere J https://orcid.org/0000-0002-1008-0687

Choisy M https://orcid.org/0000-0002-5187-6390

Chou T https://orcid.org/0000-0003-0785-6349

Choudhury A

Chouvenc T https://orcid.org/0000-0003-3154-2489

Chowdhury S

Chowell G https://orcid.org/0000-0003-2194-2251

Chung M-H

Cinar ME

Cisneros Armas D

Clairambault J

Clapham P

Clark M

Clark N

Clarté G

Clay Z https://orcid.org/0000-0002-3016-1732

Clemente C https://orcid.org/0000-0001-8174-3890

Clifton S https://orcid.org/0000-0001-5949-068X

Cobey S

Cocking C

Coguiec E https://orcid.org/0000-0002-5454-0948

Cohen E

Colebank M

Collins M

Collins R https://orcid.org/0000-0002-1714-7215

Colquhoun D https://orcid.org/0000-0002-4263-017X

Colubri A https://orcid.org/0000-0001-5559-9661

Columbus S https://orcid.org/0000-0003-1546-955X

Coomes J https://orcid.org/0000-0002-6196-5370

Cordero-Rivera A https://orcid.org/0000-0002-5087-3550

Corley M

Cornelissen P

Correa LEO

Corsetti F https://orcid.org/0000-0002-2275-436X

Cossin-Sevrin N

Costa da Silva R

Coste C

Cousseau L https://orcid.org/0000-0001-9719-0434

Crawford C https://orcid.org/0000-0002-7225-8137

Crevecoeur F

Crofts S https://orcid.org/0000-0003-1100-0009

Cronk Q https://orcid.org/0000-0002-4027-7368

Crook B

Crowe-Riddell J https://orcid.org/0000-0003-2794-2914

Croy I

Crüwell S https://orcid.org/0000-0003-4178-5820

Cruz Elizalde R

Cueva del Castillo R https://orcid.org/0000-0002-6385-0729

Culbertson J https://orcid.org/0000-0002-1737-6296

Cunningham E

Da Silva J

Dahmen D

Dal Pesco F https://orcid.org/0000-0003-2326-1185

Daly R

D'Ambrosi R

D'Amelio P https://orcid.org/0000-0002-4095-6088

Damer B

Dammhahn M

Dandekar T

Dankwa E

Darques R

Das A

Das S

Das S

Daughters K https://orcid.org/0000-0001-5889-8464

Dautriche I https://orcid.org/0000-0002-2297-985X

David-Barrett T https://orcid.org/0000-0002-8979-3136

Davies K https://orcid.org/0000-0001-6060-2394

Davis DS

Davis S

de Celis A https://orcid.org/0000-0002-7362-7360

De Groote F https://orcid.org/0000-0002-4255-8673

de Guinea M https://orcid.org/0000-0002-8825-929X

DE HEVIA MD https://orcid.org/0000-0002-1495-3721

de Laat J

de Miguel-Arribas A

De Moor D https://orcid.org/0000-0003-2474-6125

De Silva T https://orcid.org/0000-0001-5539-1617

Deaner R

DeCasien A

Dedong HU

Deeming DC https://orcid.org/0000-0002-9587-6149

Deffner D https://orcid.org/0000-0002-1649-3861

Deffuant G

Dege N

Degnan B https://orcid.org/0000-0001-7573-8518

Del Giudice M https://orcid.org/0000-0001-8526-1573

del Mar Gil Oviedo M

Delhey K https://orcid.org/0000-0001-5190-5406

DeMars L

Demartsev V https://orcid.org/0000-0002-1456-9789

DeNardo D https://orcid.org/0000-0003-3145-8367

Derstine N https://orcid.org/0000-0002-0671-7702

Deshpande A https://orcid.org/0000-0003-2581-2737

DeVito N

Dey C https://orcid.org/0000-0003-4947-8972

Dhanasekar M

Di Nunno F

Díez Díaz V https://orcid.org/0000-0002-9840-9829

Dinavahi V

Ding W

Dissegna A https://orcid.org/0000-0002-4333-3353

Dixit M

Dixit T https://orcid.org/0000-0001-5604-7965

Djanian S

Dobrovolny H https://orcid.org/0000-0003-3592-6770

Dodat P-J

Dodel M https://orcid.org/0000-0002-1724-9609

Dollion A

Dolnik M

Domínguez-García Á

Dong C

Dos Santos D

Dotterer H

Dowker A

Downing P

Dowthwaite L

Driscoll D

Droz C

Drysdale T

Du Y https://orcid.org/0000-0002-1077-382X

Dubowsky J

Duempelmann M

Dukas R https://orcid.org/0000-0003-4533-8542

Dunhill A https://orcid.org/0000-0002-8680-9163

Dunlop R https://orcid.org/0000-0002-0427-6317

Dvoriashyna M

Dyble M

Dylewsky D https://orcid.org/0000-0003-4904-1391

Dzewaltowski A https://orcid.org/0000-0003-1999-0246

Ebissa DT https://orcid.org/0000-0001-7735-9060

Edwards P https://orcid.org/0000-0003-3658-5146

Efron B

Eggeling R

Ekroos J

Eliseo-Arras R

Elsayed O https://orcid.org/0000-0002-7203-4846

Emam H

Emara S https://orcid.org/0000-0002-9126-4858

Engelmann N

Erban T

Errington T https://orcid.org/0000-0002-4959-5143

Espin Noboa LE https://orcid.org/0000-0002-3945-2966

Espín A https://orcid.org/0000-0002-5038-3877

Esterman M

Esteve J

Estevez-Soto P

Exnerova A

Ezzat L https://orcid.org/0000-0002-4317-6458

Fahrbach S

Falcó C https://orcid.org/0000-0001-9832-2697

Falkingham P

Fall M https://orcid.org/0000-0002-8340-1525

Falótico T https://orcid.org/0000-0002-0638-1989

Fan G-Y

Fan L

Fantoni F

Faraone N

Farina SC https://orcid.org/0000-0003-2479-1268

Farjam M https://orcid.org/0000-0002-0882-4851

Farrauto R

Feder J

Félix S

Feng T https://orcid.org/0000-0001-6734-0462

Fenton B https://orcid.org/0000-0001-9761-5412

Fernández-Castilla B

Ferreira MA

Ferreira VH https://orcid.org/0000-0001-7752-2382

Ferretti L

Fesce E

Fichtel C https://orcid.org/0000-0002-8346-2168

Field S https://orcid.org/0000-0001-7874-1261

Figgener C

Figueirido Castillo FB https://orcid.org/0000-0003-2542-3977

Findlater M

Finkelstein A https://orcid.org/0000-0003-3490-7228

Fischer E https://orcid.org/0000-0002-2916-0900

Fisher D https://orcid.org/0000-0002-4444-4450

Fitneva S

Flanagan S https://orcid.org/0000-0002-2226-4213

Flannery Sutherland J https://orcid.org/0000-0001-8232-6773

Fleming A https://orcid.org/0000-0003-3301-5586

Flood P

Flury R https://orcid.org/0000-0002-0349-8698

Fogel A https://orcid.org/0000-0003-3048-7959

Folbre N

Fontes F

Fonzo G

Ford J

Fornaciai M

Fortunato L https://orcid.org/0000-0001-8546-9497

Foster W https://orcid.org/0000-0002-9595-8195

Francis R

Frandsen P

Fraser N

Frasier T https://orcid.org/0000-0002-3055-0199

Freeman E

Frese M

Friberg U https://orcid.org/0000-0001-6112-9586

Fricke C

Fristrup K https://orcid.org/0000-0002-7467-9314

Froldi G

Fusaroli R

Gabor C https://orcid.org/0000-0001-7584-1451

Galli S

Gallou F

Gamble A https://orcid.org/0000-0001-5430-9124

Gandhi L https://orcid.org/0000-0001-5166-9431

Gao H https://orcid.org/0000-0001-6852-9435

García JO https://orcid.org/0000-0001-6559-0652

Garcia J https://orcid.org/0000-0002-4183-1404

Gardner A https://orcid.org/0000-0002-1304-3734

Gasparyan AY

Geffroy B https://orcid.org/0000-0001-6120-1103

Gehr B https://orcid.org/0000-0002-1044-9296

Genikhovich G https://orcid.org/0000-0003-4864-7770

Georgakarakos E

Gerken A

Geronikaki A

Gerothanassis I

Gibson B https://orcid.org/0000-0001-9620-0063

Gilbert A

Gilch S

Gillet A

Giner-Sorolla R

Gjini E https://orcid.org/0000-0002-0493-3102

Gloss A

Goblirsch M

Godoy P https://orcid.org/0000-0003-4519-5094

Goeting SHMS

Gogarten J https://orcid.org/0000-0003-1889-4113

Goharshady A https://orcid.org/0000-0003-1702-6584

Gokce A https://orcid.org/0000-0002-5032-7007

Golas B https://orcid.org/0000-0003-0568-6702

Goldsworthy A

Golnar A

Gomes S https://orcid.org/0000-0002-8731-367X

Gómez-Rubio V

Gonçalves J

Gonçalves P https://orcid.org/0000-0002-6987-4836

Goni-Moreno A https://orcid.org/0000-0002-2097-2507

Goodman S

Gordon R

Goren AC

Gotham M

Graham B https://orcid.org/0000-0001-6582-2273

Grandini CR https://orcid.org/0000-0002-3336-309X

Grassberger P

Graves T

Grecian WJ

Green A

Green J

Greenhalgh S https://orcid.org/0000-0003-2484-4147

Greer M https://orcid.org/0000-0002-9543-4498

Grenning A

Grey W

Griffiths H

Grima R https://orcid.org/0000-0002-1266-8169

Gripp Fernandes G https://orcid.org/0000-0002-4776-8346

Groh M https://orcid.org/0000-0002-9029-0157

Gross T https://orcid.org/0000-0002-1356-6690

Grove T

Gruber T

Gu J

Gu Y

Guan B

Guan YJ

Guarneri A https://orcid.org/0000-0002-9162-2731

Guddati M

Gueriau P

Guiry EJ

Gulizzi V

Gundersen OE https://orcid.org/0000-0002-9754-5941

Gupta R

Gurarie D https://orcid.org/0000-0002-5314-7888

Guruprasad L

Haaland T

Haase A https://orcid.org/0000-0002-8324-0047

Haase J

Haddock G https://orcid.org/0000-0001-5293-2772

Hahn D

Hajjarian Z

Haklay M (Muki) https://orcid.org/0000-0001-6117-3026

Halava V

Halawa M https://orcid.org/0000-0001-6358-2691

Hall J

Hallman TA

Hanley D https://orcid.org/0000-0003-0523-4335

Hanson L

Harikumar R

Harper R

Harris M https://orcid.org/0000-0001-8196-0430

Harrison JJ

Harrison L https://orcid.org/0000-0002-6690-5035

Harrison P https://orcid.org/0000-0002-9851-9462

Hartgerink C https://orcid.org/0000-0003-1050-6809

Hartlieb M

Harvey C https://orcid.org/0000-0002-2830-0844

Hasan M N

Haulsee D

Hawes C

He M

He Z

Heck D

Heckert A

Heddam S https://orcid.org/0000-0002-8055-8463

Hedin M

Hedrick T https://orcid.org/0000-0002-6573-9602

Hegre H

Heit B

Helble T

Hellmann J https://orcid.org/0000-0003-0479-3960

Hempel de Ibarra N https://orcid.org/0000-0002-0859-8217

Hendriks F

Hendrix J

Hengel E

Henry R

Henzi P https://orcid.org/0000-0001-6175-1674

Herbison R https://orcid.org/0000-0001-5335-2382

Hernandez JM https://orcid.org/0000-0002-0704-0035

Hernik M

Herrera R

Hertler C https://orcid.org/0000-0002-8252-9674

Hex S https://orcid.org/0000-0002-9142-6917

Heyman T https://orcid.org/0000-0003-0565-441X

Hifzhudin Noor Aziz M

Higginson A

Hill E https://orcid.org/0000-0002-2992-2004

Hillen T

Ho Y-S

Hockings K

Hoffmann S https://orcid.org/0000-0001-6197-8801

Holick M

Holl P

Holland R https://orcid.org/0000-0003-4495-8061

Holtz T

Hong Z https://orcid.org/0000-0002-5343-3008

Hopkins R

Hoque SM https://orcid.org/0000-0002-4233-9374

Hortobagyi T

Hosack G

Hosseinzadeh G

Houston-Price C

Hrncir M https://orcid.org/0000-0003-4931-3924

Hrushka D

Hsieh C-H

Hsu Y-J

Hu D https://orcid.org/0000-0002-0017-7303

Huang W

Hübler N https://orcid.org/0000-0002-0013-563X

Huma B https://orcid.org/0000-0003-0482-9580

Hunt T

Hunter E https://orcid.org/0000-0002-1767-4744

Hunter L

Hunyadi B

Hurst G

Hurtado N https://orcid.org/0000-0002-2740-3745

Hutchins K

Ichihara M https://orcid.org/0000-0002-5591-7129

Ichinose T

Indeck K https://orcid.org/0000-0002-0956-5070

Innominato P

Ionel I https://orcid.org/0000-0002-2999-1210

Isager P https://orcid.org/0000-0002-6922-3590

Ivandini TA https://orcid.org/0000-0002-5282-5325

Ivimey-Cook E https://orcid.org/0000-0003-4910-0443

Ivory J

Ivry R

Iwaniuk A https://orcid.org/0000-0001-9273-3655

Jabbari S https://orcid.org/0000-0001-5235-0406

Jablonski D

Jaeger B

Jagtap S https://orcid.org/0000-0002-6425-7118

Jahanbakhshi A

James A https://orcid.org/0000-0003-2859-7796

James L https://orcid.org/0000-0002-9600-4473

Janisch J https://orcid.org/0000-0003-3300-6292

Jaramillo A

Javed K

Jeanneret R https://orcid.org/0000-0002-9444-9129

Jedlikowski J

Jenkins D

Jenkins E

Jensen K

Jessopp M https://orcid.org/0000-0002-2692-3730

Ji Y

Jiang B

Jiang W https://orcid.org/0000-0003-0953-5047

Jiang X

Jin NL https://orcid.org/0000-0003-1267-7396

Jin Y

Joglekar Y

Johnson M

Johnson M

Johnson MA https://orcid.org/0000-0002-9278-5639

Jones B https://orcid.org/0000-0001-7777-0220

Jones DA

Jones J

Josic K

Joyce J

Kaan E

Kabani M https://orcid.org/0000-0001-7440-6394

Kabir KM https://orcid.org/0000-0003-0249-5417

Kabla A

Kafarski P

Kaiser D https://orcid.org/0000-0002-9007-3160

Kalita G

Kambey CSB

Kamgang V

Kammerer C https://orcid.org/0000-0002-0596-623X

Kamran U

Kang K https://orcid.org/0000-0003-1230-3626

Kang R

Kantz H

Kao A https://orcid.org/0000-0001-8232-8365

Kaplan G

Kappel Sarah

Kappeler P https://orcid.org/0000-0002-4801-487X

Karczmarski L

Karl J

Karsdorp F https://orcid.org/0000-0002-5958-0551

Karwinkel T https://orcid.org/0000-0001-6433-3013

Katsube M

Kaul C

Kavanagh K https://orcid.org/0000-0002-1496-990X

Kavé G

Kawabe S

Kawaguchi Y

Kaya Y https://orcid.org/0000-0003-1506-7913

Keeler A

Kegode TM https://orcid.org/0000-0002-6888-5541

Kempraj V https://orcid.org/0000-0002-6151-8618

Kempton J https://orcid.org/0000-0002-4763-8066

Kendig M https://orcid.org/0000-0003-3337-6402

Kennedy L https://orcid.org/0000-0002-9067-0221

Kenney-Lazar M

Kentner G

Kenward B

Khan I

Khanday WA

Khassetarash A

Kibbe M

Kibler A

Kibuchi E

Kieslich K

Kim s

Kim Y https://orcid.org/0000-0001-6731-7123

Kimmig J https://orcid.org/0000-0001-8032-4272

Kimmitt A https://orcid.org/0000-0003-0044-8297

King A

Kirsch D

Kistnova E

Klapwijk E https://orcid.org/0000-0002-8936-0365

Klug C https://orcid.org/0000-0002-4099-7453

Kluger A

Knorre D https://orcid.org/0000-0001-7468-6963

Kobayashi K https://orcid.org/0000-0002-9475-6807

Kobayashi N

Koelewijn https://orcid.org/0000-0002-1867-0374

Kohno Y

Koirala D https://orcid.org/0000-0001-6424-3173

Komata S https://orcid.org/0000-0001-7231-6344

Konecka E

Koroteev PS https://orcid.org/0000-0001-8484-9201

Koskinen I https://orcid.org/0000-0002-9060-7011

Köster G

Köster M

Koutstaal W https://orcid.org/0000-0003-2464-6511

Kowialiewski B https://orcid.org/0000-0002-7743-761X

Kozakis T https://orcid.org/0000-0002-3868-2129

Kramer P

Krams I https://orcid.org/0000-0001-7150-4108

Kranz C

Krasheninnikova A https://orcid.org/0000-0001-8566-8277

Krasteva V

Krauel J https://orcid.org/0000-0003-2207-8952

Krill J

Krishnan A https://orcid.org/0000-0002-8317-4991

Krishnaswamy J https://orcid.org/0000-0001-7985-0005

Kriwet J

Krueger J

Krukar J

Krull M

Kruzan KP

Kulahci I https://orcid.org/0000-0003-0104-0365

Kundu S

Kuperman M

Kurasaki M

Kurokawa S

Kuzmin E

Kuznetsov A

Labarga M

Lacerda CMR

Lachmann M

Ladosz P

Lagaňa R

Lainé N

Lakens D https://orcid.org/0000-0002-0247-239X

Lamb J

Lambert J-F

Lam-Gordillo O

Lamont M

Lane C

Lanfranco R

Lang A

Lang M https://orcid.org/0000-0003-2604-4733

Langelaan D

Lany J

Laohakunakorn N

Laporta G

Larouche O

Larsen PS https://orcid.org/0000-0002-7155-5965

Larsen S

LaSharr TN

Lavan N

Lavesque N

Le D

Le M-V https://orcid.org/0000-0002-6651-0121

Lea S https://orcid.org/0000-0002-8498-4187

Leblanc A

LebretonM

Lee KP https://orcid.org/0000-0001-9770-0078

Lee P

Lehman J

Lemaire P

Lemell P

Lente G

Lenz P

Leonard D

Leontaris G

Lesaffre T https://orcid.org/0000-0002-0815-259X

Lesch R https://orcid.org/0000-0001-7151-252X

Leslie D

Lessios H

Leu M-Y

Levesque D https://orcid.org/0000-0003-0132-8094

Lewandowsky S

Lewinson O

Lewis R https://orcid.org/0000-0003-3533-6105

Li A

Li L https://orcid.org/0000-0002-2447-6295

Li T https://orcid.org/0000-0002-7495-8022

Li X

Liang W https://orcid.org/0000-0002-0004-9707

Lieck D https://orcid.org/0000-0001-9814-3488

Liger-Belair G

Ligocki I

Lim J

Ling A

List F

Liu H

Liu L https://orcid.org/0000-0003-0286-8885

Liu N

Liu R

Liu S-X https://orcid.org/0000-0001-6104-4320

Liu X

Lobbia P

Lockley M

Lockwood PL https://orcid.org/0000-0001-7195-9559

Loconsole M https://orcid.org/0000-0002-0024-2670

Long G

Long RA

Longo M https://orcid.org/0000-0002-2450-4903

Lopes R https://orcid.org/0000-0003-2193-5107

López J

López-Parra AM

Lopez-Urias F

Lötters S

Lou Y https://orcid.org/0000-0003-3864-2001

Lovas-Kiss Á https://orcid.org/0000-0002-8811-1623

Lowe G

Lowy D

Loy J

Lu H (Yvonne)

Lubensky D

Lübken M

Luboobi L

Lucas J https://orcid.org/0000-0001-9661-7985

Luchiari A https://orcid.org/0000-0003-3294-7859

Lucon-Xiccato T https://orcid.org/0000-0002-1017-9230

Luscher A

Lustri L https://orcid.org/0000-0002-8951-5160

Lv H https://orcid.org/0000-0002-9212-7572

Lymbery R https://orcid.org/0000-0001-8041-6169

Lynch V

Campos C https://orcid.org/0000-0002-5151-6774

Ma Y

Macartney E https://orcid.org/0000-0003-3866-143X

Macías-García C https://orcid.org/0000-0003-3242-4214

MacLaren J https://orcid.org/0000-0003-4177-227X

Magana A

Mahdy A https://orcid.org/0000-0003-2218-5408

Maio G

Majtán J

Malani A

Malatesta G https://orcid.org/0000-0002-7658-2364

Malcolm S

Mammarella I

Manataki A

Mandal D

Manjarrez E

Mann R https://orcid.org/0000-0003-0701-1274

Mansfield A

Manzone D

Mar K

Marciniak-Czochra A https://orcid.org/0000-0002-5831-6505

Marley S

Maroja L

Marone L

Marozas V

Marshall HH

Martelli S

Martin A https://orcid.org/0000-0002-2843-6928

Martin B

Martin J

Martin R

Martinez L

Martinig AR https://orcid.org/0000-0002-0972-6903

Martinson W https://orcid.org/0000-0002-3590-606X

Martis J E

Martisius N

Marzban S https://orcid.org/0000-0002-9435-017X

Maselli A

Maslov S

Mason R

Masonick P

Massey A

Maszaros MS

Mathis A

Mathpal MC

Matsuyama A https://orcid.org/0000-0002-6135-6276

Mauri E https://orcid.org/0000-0001-8100-3956

May Concha I

Mazza E

Mbewe J

McCabe B

McCabe R https://orcid.org/0000-0001-8649-6913

McCartney M

McChesney H https://orcid.org/0000-0002-9981-1342

McCracken G https://orcid.org/0000-0001-9017-4079

McCullough JLK

McCune K https://orcid.org/0000-0003-0951-0827

McFeeters B

McGowan C

McNeely-White K

McPherson J

McQuillan M

Medina-Ortiz D

Mehr S https://orcid.org/0000-0002-9400-7718

Melica V https://orcid.org/0000-0002-1820-3418

Melita D https://orcid.org/0000-0003-2703-6735

Mengarelli A

Merlino A

Metcalfe N https://orcid.org/0000-0002-1970-9349

Meyer CA

Meyer D https://orcid.org/0000-0003-0050-6354

Middleton R

Mideo N

Mihret Aragaw A

Millard A

Millard M

Miller III D

Miller B

Miller C https://orcid.org/0000-0003-0758-9447

Miller JA

Miller N https://orcid.org/0000-0001-9363-9919

Mingorance J https://orcid.org/0000-0001-6173-5711

Mingotti A

Minton G

Miranda E https://orcid.org/0000-0003-2198-4742

Mirkarimi P https://orcid.org/0000-0003-1796-0916

Mitrovic M

Mittelstadt B

Miyoshi D

Mizumoto N https://orcid.org/0000-0002-6731-8684

Mladineo I https://orcid.org/0000-0003-1364-3593

Mobley K

Modrák M 02-8886-7797

Mognetti B

Mohring B https://orcid.org/0000-0001-8553-4041

Molho C https://orcid.org/0000-0002-2846-2544

Molina-Morales M

Molinari B

Molleman F https://orcid.org/0000-0002-6551-266X

Molteni F

Monadjem A https://orcid.org/0000-0003-1906-4023

Monarrez PM

Montaggioni L

Montgomerie R https://orcid.org/0000-0003-4701-4525

Monti F

Moore M

Morelli F https://orcid.org/0000-0003-1099-1357

Moreno García C

Morgane T

Morris M https://orcid.org/0000-0001-9962-2982

Morsky B

Mouginot P https://orcid.org/0000-0003-2967-065X

Mounce R

Mukherjee S https://orcid.org/0000-0002-2974-4447

Müller R https://orcid.org/0000-0002-4688-1515

Mullish B https://orcid.org/0000-0001-6300-3100

Mummadisetti M

Munn A

Murakami H https://orcid.org/0000-0002-1433-0524

Murakami M

Murray E

Murugavel B https://orcid.org/0000-0002-5130-9935

Musa R

Musa S https://orcid.org/0000-0001-6335-2335

Mushayabasa S https://orcid.org/0000-0002-7423-2934

Nabati P

Nagano H

Nagarale R

Naggar YAl

Nagy L

Nakagawa S https://orcid.org/0000-0002-7765-5182

Nakata T https://orcid.org/0000-0002-0378-2242

Nalon E

Nambiar S

Nareddy VR https://orcid.org/0000-0001-6840-2397

Nasari H

Näslund J

Nasr JJ https://orcid.org/0000-0002-2423-7315

Navazani S

Naya D https://orcid.org/0000-0002-8311-9263

Neto V

Newlands NK

Ng, Eddie YK

Ngcamphalala C

Nguyen N

Nielsen RKL

Nilsonne G https://orcid.org/0000-0001-5273-0150

Niranjan M

Nitsch F https://orcid.org/0000-0002-7832-7498

Nohejlova M

Noor D

Northridge S

Noskov A https://orcid.org/0000-0003-0503-6558

Nuijten M

Nussbaum C

Odgers C

Odriozola JA https://orcid.org/0000-0002-8283-0459

Ogawa M https://orcid.org/0000-0002-3781-2016

O'Hara M

Okada Y https://orcid.org/0000-0003-4528-2980

Okafor E

O'Keefe J

Oleszkiewicz A https://orcid.org/0000-0003-2217-1858

Oliveira HPde

Oliver S https://orcid.org/0000-0002-2975-1233

Oosthuizen C

Oosthuizen M https://orcid.org/0000-0001-6305-8283

Ortega-Ramos PA https://orcid.org/0000-0003-3339-2410

Orton D

Orubuloye O

Osiejuk T

Oster H

Ostojic L

Otero M

Ottoni E https://orcid.org/0000-0003-1288-8345

Outeda P

Owusu Boadi N

Paiva V

Pal A https://orcid.org/0000-0001-7806-2453

Pal M

Palacios-Gimenez OM https://orcid.org/0000-0002-1472-9949

Pale U

Palottini F

Palus M https://orcid.org/0000-0001-8474-1436

Papadopoulos K https://orcid.org/0000-0003-1042-6016

Papadopoulou M https://orcid.org/0000-0002-6478-8365

Papastamatiou Y https://orcid.org/0000-0002-6091-6841

Paquet P

Park H

Parker J

Parmar AS

Parmar S https://orcid.org/0000-0003-0088-518X

Parnamets P

Parslew B https://orcid.org/0000-0002-0070-488X

Pasch B

Pasetto D https://orcid.org/0000-0001-6892-9826

Patel H

Patterson S https://orcid.org/0000-0002-5283-8919

Paul S

Paulus Y

Pavez-Fox M https://orcid.org/0000-0001-5097-2500

Peckre L

Peng F https://orcid.org/0000-0002-1637-5611

Peng Q

Pensalfini M https://orcid.org/0000-0003-3296-9388

Pentlavalli S

Pereg M

Perez-Rosales G https://orcid.org/0000-0001-6577-3416

Perini F

Perlmutter J

Pessoa DMA https://orcid.org/0000-0002-2516-6766

Peter OJ

Petermann H https://orcid.org/0000-0002-3847-2244

Peters K

Petralia S

Petsios E https://orcid.org/0000-0002-7955-7412

Phani V

Pickering J https://orcid.org/0000-0002-7242-9207

Pickle N

Picu C

Piechaud N

Piličiauskas G

Pillar V

Pineda M

Pineiro M https://orcid.org/0000-0002-7460-3758

Pinfield S https://orcid.org/0000-0003-4696-764X

Pinheiro A https://orcid.org/0000-0002-7981-3682

Pinto Neto O

Pirola S

Plank M https://orcid.org/0000-0002-7539-3465

Pohlmann K

Polhill G https://orcid.org/0000-0002-8596-0590

van Poorten B T v

Porffy L

Porter E https://orcid.org/0000-0002-9530-6955

Portnoy D

Portugal S https://orcid.org/0000-0002-2438-2352

Post S https://orcid.org/0000-0003-1091-9742

Potestio R https://orcid.org/0000-0001-6408-9380

Poulsen M https://orcid.org/0000-0002-2839-1715

Poust A https://orcid.org/0000-0001-7955-613X

Prasad S

Prather M

Preedy K https://orcid.org/0000-0002-9286-5364

Pregler K

Preston E https://orcid.org/0000-0002-4098-2630

Prike T https://orcid.org/0000-0001-7602-4947

Prosser D

Puranik A

Quigley C https://orcid.org/0000-0002-7522-4426

Quinn T

Qureshi A https://orcid.org/0000-0002-7698-2691

Qureshi A

Rafique U

Raghu AV https://orcid.org/0000-0003-0299-7409

Raghunathan S

Rahim F

Rahman I https://orcid.org/0000-0001-6598-6534

Rahman M M

Raia P https://orcid.org/0000-0002-4593-8006

Rainforth M

Ramachandra V

Ramirez A

Ramirez FJ

Ramirez P

Ranzini M

Rao KSVK

Raška J

Rast W

Rault A

Ravinetto R https://orcid.org/0000-0001-7765-2443

Raynaud F

Read A

Reber S

Reddin C https://orcid.org/0000-0001-5930-1164

Reed R

Refai MIM

Reimchen T

Reimer J https://orcid.org/0000-0003-0453-8804

Rein R

Reina A https://orcid.org/0000-0003-4745-992X

Reinhold I https://orcid.org/0000-0001-6519-3809

Reinhold L https://orcid.org/0000-0002-1168-9160

Reisz R https://orcid.org/0000-0002-7454-1649

Remnant E https://orcid.org/0000-0002-3808-8365

Reynvoet B

Ribas C

Ricci F https://orcid.org/0000-0003-2501-6925

Richards H

Richards J https://orcid.org/0000-0002-0697-2551

Richardson P https://orcid.org/0000-0001-9115-2445

Richiardi M https://orcid.org/0000-0002-3749-7386

Ridgely R

Riegel M

Rimbach R https://orcid.org/0000-0003-3059-0382

Rippon G

Robbins M

Roberts A https://orcid.org/0000-0003-1185-7897

Roberts S https://orcid.org/0000-0001-5990-9161

Robinson E https://orcid.org/0000-0002-1299-6541

Robinson J

Robson S

Rodrigues A https://orcid.org/0000-0003-3231-551X

Rodrigues F https://orcid.org/0000-0002-0145-5571

Rohlfs M https://orcid.org/0000-0002-8767-1629

Römbke T

Rong Z

Roopnarine P https://orcid.org/0000-0002-9811-1176

Root-Gutteridge H https://orcid.org/0000-0001-9854-2948

Roozenbeek J https://orcid.org/0000-0002-8150-9305

Rose P

Röseler L

Ross-Hellauer T https://orcid.org/0000-0003-4470-7027

Rossi F

Rossi N https://orcid.org/0000-0003-0780-7117

Rößler D https://orcid.org/0000-0003-4678-2231

Rostaher A

Rota E https://orcid.org/0000-0002-8235-6419

Rowell M

Rowland S

Roy S

Rub MA

Rubin M https://orcid.org/0000-0002-6483-8561

Rueda N

Ruppert D

Russell A

Russo D

Russo N

Ruta M

Ruthrauff DR https://orcid.org/0000-0003-1355-9156

Ryan C

Ryan J

Rybak JM

Rzadkowski G https://orcid.org/0000-0002-8238-2481

Sadeh S

Sahasranaman A https://orcid.org/0000-0002-9613-8041

Sahraoui H

Saielli G

Saint-Aubin J

Sakiyama T

Salazar-Hamm P https://orcid.org/0000-0002-6168-5723

Saleem S

Salje E

Sallan L

Saltelli A https://orcid.org/0000-0003-4222-6975

Saltzman W

Samaan MA

Samaniego H https://orcid.org/0000-0002-2485-9827

Sameer M

Samtani S

Samuel S

Sanchez Garcia R

Sanchez Herrera M https://orcid.org/0000-0001-5144-4996

Sánchez-Tójar A https://orcid.org/0000-0002-2886-0649

Sander PM

Sandrine G

Sanjay V

Sankey DWE https://orcid.org/0000-0002-6363-8023

Sans V

Santoni D

Saracco F

Sarafoglou A

Satoh S https://orcid.org/0000-0003-1284-5249

Savi T https://orcid.org/0000-0001-7585-763X

Scanlan P https://orcid.org/0000-0002-6733-9653

Schaeffer R https://orcid.org/0000-0001-7930-6878

Schall E https://orcid.org/0000-0002-7740-5466

Scheffer M

Schelsky W https://orcid.org/0000-0002-5742-757X

Scheun J

Scheyer T https://orcid.org/0000-0002-6301-8983

Schlupp I https://orcid.org/0000-0002-2460-5667

Schmal M

Schmalz X

Schmid D

Schmidt J https://orcid.org/0000-0002-0412-396X

Schmitt P https://orcid.org/0000-0001-8455-952X

Schneditz D https://orcid.org/0000-0002-0124-1012

Schneider J https://orcid.org/0000-0001-8523-7354

Schneider-Crease I

Schönbrodt F

Schripsema J

Schroeder J https://orcid.org/0000-0002-0538-0730

Schult J

Schultner E https://orcid.org/0000-0002-5069-9732

Schultze A

Schwarb H

Schwarz C

Schwarzkopf DS https://orcid.org/0000-0003-3686-1622

Scott R

Scrivener C

Scullin M

Sealy J

Sear R https://orcid.org/0000-0002-4315-0223

Segalini A https://orcid.org/0000-0001-8667-0520

Segura V

Selden P

Semkovska M

Sena M https://orcid.org/0000-0003-4708-999X

Senior A https://orcid.org/0000-0001-9805-7280

Senulis A https://orcid.org/0000-0002-4759-2707

Sergio F

Serrano E

Seyfried A

Shabangu FW https://orcid.org/0000-0002-3668-3566

Shabbir W https://orcid.org/0000-0002-0753-7850

Shackell N

Shadwick R

Shahabi C

Shailk A

Sharp A https://orcid.org/0000-0001-5117-5335

Sheehan M https://orcid.org/0000-0002-3949-7873

Sheikhhassani R

Shelke N

Sheridan J

Shestopaloff A

Shi L

Shimizu K https://orcid.org/0000-0001-6811-5275

Shortall C https://orcid.org/0000-0002-7175-5393

Shruthi J

Shu D

Shu L https://orcid.org/0000-0002-8122-334X

Shukla P https://orcid.org/0000-0002-4664-4130

Siebert MJ https://orcid.org/0000-0003-4385-5773

Silk M https://orcid.org/0000-0002-8318-5383

Silver AM

Simani S

Simbeck K

Simons D https://orcid.org/0000-0001-9655-1656

Simpson S

Sims NA

Singh A https://orcid.org/0000-0003-3012-5245

Siniscalchi M https://orcid.org/0000-0002-1592-6549

Sinopoli M

Siu-Wing Chan A

Siviglia A

Skoronski E

Skubalska-Rafajlowicz E https://orcid.org/0000-0002-2795-3835

Sliney D

Smerdon D

Smith D

Smith DL

Smith E

Smith M https://orcid.org/0000-0001-5660-1727

Smith-Aguilar SE https://orcid.org/0000-0003-2209-5496

Smolka M

Sniegula S https://orcid.org/0000-0003-1459-3751

Sobral G https://orcid.org/0000-0003-2858-3669

Sofonea M

Sohng JK

Sojkova M

Solé R https://orcid.org/0000-0001-6974-1008

Soleimani M

Song X

Song Y https://orcid.org/0000-0002-9525-2870

Sonne J https://orcid.org/0000-0002-8570-7288

Sookias R https://orcid.org/0000-0002-5189-4011

Soorati M

Soto D

Sotola V

Souza MSDe

Sovrano F https://orcid.org/0000-0002-6285-1041

Spedding G http://orcid.org/0000-0003-3033-7897

Speekenbrink M https://orcid.org/0000-0003-3221-1091

Speight I

Spence A

Spiegel O https://orcid.org/0000-0001-8941-3175

Spiridonov A

Spitzen J https://orcid.org/0000-0003-0455-7511

Sportelli J

Srivathsan A

Staub K

Stefanaki A https://orcid.org/0000-0002-6393-9416

Stepney S https://orcid.org/0000-0003-3146-5401

Stift M https://orcid.org/0000-0001-7801-9498

Stock M https://orcid.org/0000-0003-0903-6061

Stock S https://orcid.org/0000-0002-9517-7970

Stockmaier S https://orcid.org/0000-0001-8280-8086

Stoffel M

Stojadinović B

Stone A

Storchi R https://orcid.org/0000-0002-3656-7514

Straley J

Strand J

Strauss E https://orcid.org/0000-0003-3413-1642

Stritih-Peljhan N

Strömbom D https://orcid.org/0000-0002-9564-2529

Subramanian B

Subramanian V

Subramaniyam NP

Sudarsan N https://orcid.org/0000-0003-0869-5794

Sudo M

Sudyka J

Sues H-D https://orcid.org/0000-0002-9911-7254

Suhendi E

Sukmasuang R

Sumner S https://orcid.org/0000-0003-0213-2018

Sun C

Sun Z

Surapaneni V https://orcid.org/0000-0002-6241-9048

Surian L

Suweis S

Swaney W https://orcid.org/0000-0002-5065-119X

Sweetman J https://orcid.org/0000-0002-2829-1503

Szederkényi G

Taboada S

Taborsky M https://orcid.org/0000-0002-1357-4316

Taguchi M

Takács K https://orcid.org/0000-0001-9126-3233

Takahashi A https://orcid.org/0000-0002-8391-7417

Tałanda M http://orcid.org/0000-0003-3358-9539

Tamborrino M https://orcid.org/0000-0002-4661-8071

Tamura T

Tan A

Tan Q

Tandon R

Tassier T

Täuber S https://orcid.org/0000-0003-2859-1474

Tausch N

Tavassoly I

Taylor D

Taylor R https://orcid.org/0000-0002-2739-6944

Taylor W

Teichroeb J https://orcid.org/0000-0002-0908-156X

Teilmann J https://orcid.org/0000-0002-4376-4700

Teixeira P

Tello-Aburto R https://orcid.org/0000-0001-8806-5762

Templeton A http://orcid.org/0000-0001-8611-3812

Terhune D

Terraneo T

Tett A

Thareja P

Thibault R

Thiery A

Thom G https://orcid.org/0000-0001-6200-0565

Thomas J

Thomas O

Thompson González N

Thompson N https://orcid.org/0000-0002-9273-3636

Thornton A

Thorogood R https://orcid.org/0000-0001-5010-2177

Thyrring J https://orcid.org/0000-0002-1029-3105

Tilic E https://orcid.org/0000-0003-0463-322X

Tillem S

Timmermans M https://orcid.org/0000-0002-5024-9053

Tirindelli R

Tizzoni M https://orcid.org/0000-0001-7246-2341

Tobajas J

Todorov O https://orcid.org/0000-0002-0295-7557

Toftness A https://orcid.org/0000-0002-7212-8615

Togoli I

Tonleu Temgoua RC https://orcid.org/0000-0003-1876-2962

Topper T

Torok P

Torres I

Tortora L

Tournier L

Toyokawa W https://orcid.org/0000-0001-8558-8568

Toz M

Tridane A https://orcid.org/0000-0001-8471-7807

Trinajstic K

Trivellone V https://orcid.org/0000-0003-1415-4097

Trumbo S

Tu H

Tuckerman L

Tumlison R

Turjeman S

Turner W https://orcid.org/0000-0002-0302-1646

Tweedy J

Tytell E

Ulpts S

Urynbassarova D

Utsumi S

Uwe M

Vad J

Vafa F

Van Aardt A

van den Bogert T https://orcid.org/0000-0002-3791-3749

van den Heuvel J

van der Burg C https://orcid.org/0000-0002-2337-5935

van der Marel A https://orcid.org/0000-0003-3942-8314

van der Weiden A

van Guyse J

van Zelst V

Vanderelst D

Vartanian L

Vaughan-Johnston T

Vazire S https://orcid.org/0000-0002-3933-9752

Veasey J https://orcid.org/0000-0002-4989-061X

Velarde D

Verbist G

Verboom W

Vermeulen E

Vernouillet A https://orcid.org/0000-0003-2525-2936

Versace E

Veyrunes F

Vidkjær NH https://orcid.org/0000-0001-6383-7674

Vilanova E

Villafaña J

Vinauger C

Vincze O

Vinn O https://orcid.org/0000-0002-2889-2544

Virgin L https://orcid.org/0000-0001-5990-0722

Vladu IC https://orcid.org/0000-0003-3301-1112

Vogel T

Vohland K

Vold K

Volkoff H https://orcid.org/0000-0003-1344-3428

Wacewicz S https://orcid.org/0000-0003-1488-6220

Wachs J https://orcid.org/0000-0002-9044-2018

Wade L https://orcid.org/0000-0002-9973-9934

Wadee MA https://orcid.org/0000-0001-6025-0587

Wali F

Wallau G

Waller R

Walsh V

Walter J https://orcid.org/0000-0003-2983-751X

Wan A

Wang D

Wang L

Wang Q

Wang X

Wantiez L

Ward M

Warne D https://orcid.org/0000-0002-9225-175X

Wassmer T

Watanabe S

Watanabe YY

Watson D

Watson G

Webb G

Weihmann T https://orcid.org/0000-0002-6628-1816

Weinmann AE https://orcid.org/0000-0002-7999-0486

Weinrich M

Weissbrod L https://orcid.org/0000-0003-3401-8180

Welch KC

Weston M

Whelan C https://orcid.org/0000-0001-7511-2603

White R

Whitehead H https://orcid.org/0000-0001-5469-3429

Wichmann S https://orcid.org/0000-0002-3257-3087

Wijayanto I

Wijeratne P

Wilhelm E

Wilkinson D https://orcid.org/0000-0002-0348-5432

Wilkinson G https://orcid.org/0000-0001-7799-8444

Willadsen M

Willems E

Williams B https://orcid.org/0000-0002-3618-2788

Willwacher S

Wilson R https://orcid.org/0000-0003-3177-0107

Wilson S https://orcid.org/0000-0002-6282-4772

Wimmer M

Windberger U

Windmill J https://orcid.org/0000-0003-4878-349X

Winkler L

Winsberg E

Wolf L

Wolf Y

Wolfgang Schubert T

Wolfhagen J

Wolfinger N

Wolfner M

Wong F https://orcid.org/0000-0002-2309-8835

Wu A https://orcid.org/0000-0002-1801-5716

Wu F

Wuensch M

Xiao LY https://orcid.org/0000-0003-0709-0777

Xu G

Xu W

Yamauchi Y

Yanco S

Yang F

Yang G

Yanik J

Yapici K

Yates AM

Yen J

Yin C

Yin Y

Yin Z https://orcid.org/0000-0002-9391-0446

Yon D https://orcid.org/0000-0002-5511-3884

Young A https://orcid.org/0000-0003-0560-6549

Young J https://orcid.org/0000-0001-5358-9339

Yousif S

Yusa Y https://orcid.org/0000-0001-5743-3657

Zacchetti L https://orcid.org/0000-0003-3179-7473

Zachreson C https://orcid.org/0000-0002-0578-4049

Zajitschek S https://orcid.org/0000-0003-4676-9950

Zakerzadeh R

Zambo D

Zamora-Camacho F https://orcid.org/0000-0001-5485-347X

Zanchi S

Zarrouk A

Zarzeczna N https://orcid.org/0000-0002-6107-2660

Zenil H https://orcid.org/0000-0003-0634-4384

Zhai S

Zhan G https://orcid.org/0000-0002-6337-3758

Zhang H https://orcid.org/0000-0002-1241-0088

Zhang J https://orcid.org/0000-0001-7107-4814

Zhang J

Zhang, J

Zhang N

Zhang Y https://orcid.org/0000-0001-5173-562X

Zhao Y

Zheng H https://orcid.org/0000-0001-8499-0694

Zheng K https://orcid.org/0009-0007-0470-5699

Zhong J

Zhou R

Zhu H

Zhu M

Zhu X-S

Zhuravlev, A https://orcid.org/0000-0003-1611-6916

Žibert J

Ziker J https://orcid.org/0000-0001-9059-5594

Zipple MN https://orcid.org/0000-0003-3451-2103

Zrncic S

